# Interaction of Diet/Lifestyle Intervention and TCF7L2 Genotype on Glycemic Control and Adiposity among Overweight or Obese Adults: Big Data from Seven Randomized Controlled Trials Worldwide

**DOI:** 10.34133/2021/9897048

**Published:** 2021-11-03

**Authors:** Tao Huang, Zhenhuang Zhuang, Yoriko Heianza, Dianjianyi Sun, Wenjie Ma, Wenxiu Wang, Meng Gao, Zhe Fang, Emilio Ros, Liana C. Del Gobbo, Jordi Salas-Salvadó, Miguel A. Martínez-González, Jan Polak, Markku Laakso, Arne Astrup, Dominique Langin, Jorg Hager, Gabby Hul, Torben Hansen, Oluf Pedersen, Jean-Michel Oppert, Wim H. M. Saris, Peter Arner, Montserrat Cofán, Sujatha Rajaram, Jaakko Tuomilehto, Jaana Lindström, Vanessa D. de Mello, Alena Stancacova, Matti Uusitupa, Mathilde Svendstrup, Thorkild I. A. Sørensen, Christopher D. Gardner, Joan Sabaté, Dolores Corella, J. Alfredo Martinez, Lu Qi

**Affiliations:** ^1^Department of Epidemiology & Biostatistics, School of Public Health, Peking University, China; ^2^Department of Global Health, School of Public Health, Peking University, China; ^3^Key Laboratory of Molecular Cardiovascular Sciences Ministry of Education, China; ^4^Global Health Institute Peking University, China; ^5^Department of Epidemiology, School of Public Health and Tropical Medicine, Tulane University, New Orleans, LA, USA; ^6^Department of Nutrition, Harvard T.H. Chan School of Public Health, Boston, MA, USA; ^7^Department of Endocrinology & Nutrition, Institut d’Investigacions Biomèdiques August Pi Sunyer, Hospital Clínic, Barcelona, Spain; ^8^CIBER Fisiopatologia de la Obesidad y Nutrición (CIBERobn), Instituto de Salud Carlos III (ISCIII), Madrid, Spain; ^9^Stanford Prevention Research Center, Stanford University, Stanford CA, USA; ^10^Human Nutrition Unit, Faculty of Medicine and Health Sciences, Pere Virgili Health Research Institute, Rovira i Virgili University, Reus, Spain; ^11^University of Navarra, Department of Preventive Medicine and Public Health, Medical School & IDISNA, Pamplona, Spain; ^12^Department for the Study of Obesity and Diabetes, Third Faculty of Medicine, Charles University, Prague, Czech Republic; ^13^Department of Medicine, University of Eastern Finland, Kuopio, Finland; ^14^University of Copenhagen, Department of Nutrition, Exercise and Sports, Faculty of Science, Copenhagen, Denmark; ^15^Institut National de la Santé et de la Recherche Médicale (Inserm), UMR1048, Institute of Metabolic and Cardiovascular Diseases, University of Toulouse and Paul Sabatier University, Toulouse, France; ^16^Nestlé Institute of Health Sciences, Lausanne, Switzerland; ^17^Department of Human Biology, NUTRIM School of Nutrition and Translational Research in Metabolism, Maastricht University Medical Centre +, Maastricht, Netherlands; ^18^Section of Metabolic Genetics, Novo Nordisk Foundation Center for Basic Metabolic Research, Faculty of Health and Medical Sciences, University of Copenhagen, Copenhagen, Denmark; ^19^Sorbonne Université, Institute of Cardiometabolism and Nutrition (ICAN), Department of Nutrition, Pitié-Salpêtrière Hospital, Assistance Publique-Hôpitaux de Paris (AP-HP), Paris, France; ^20^Department of Medicine, Unit for Endocrinology and Diabetes, Karolinska University Hospital, Stockholm, Sweden; ^21^School of Public Health, Loma Linda University, Loma Linda, CA, USA; ^22^Department of Chronic Disease Prevention, Finnish National Institute for Health and Welfare, HelsinkiFinland; ^23^Department of Public Health, University of Helsinki, Helsinki, Finland; ^24^Diabetes Research Group, King Abdulaziz University, Jeddah, Saudi Arabia; ^25^Institute of Public Health and Clinical Nutrition, University of Eastern Finland, Kuopio, Finland; ^26^Danish Diabetes Academy Odense, Denmark; ^27^Department of Public Health, Section of Epidemiology, Faculty of Health and Medical Sciences, University of Copenhagen, Denmark; ^28^Department of Preventive Medicine, University of Valencia, Valencia, Spain; ^29^Department of Nutrition Food Science and Physiology, University of Navarra, IDISNA, Pamplona and IMDEA, Madrid, Spain

## Abstract

*Objective*. The strongest locus which associated with type 2 diabetes (T2D) by the common variant rs7903146 is the transcription factor 7-like 2 gene (*TCF7L2*). We aimed to quantify the interaction of diet/lifestyle interventions and the genetic effect of *TCF7L2* rs7903146 on glycemic traits, body weight, or waist circumference in overweight or obese adults in several randomized controlled trials (RCTs).*Methods*. From October 2016 to May 2018, a large collaborative analysis was performed by pooling individual-participant data from 7 RCTs. These RCTs reported changes in glycemic control and adiposity of the variant rs7903146 after dietary/lifestyle-related interventions in overweight or obese adults. Gene treatment interaction models which used the genetic effect encoded by the allele dose and common covariates were applicable to individual participant data in all studies.*Results*. In the joint analysis, a total of 7 eligible RCTs were included (n=4,114). Importantly, we observed a significant effect modification of diet/lifestyle-related interventions on the *TCF7L2* variant rs7903146 and changes in fasting glucose. Compared with the control group, diet/lifestyle interventions were related to lower fasting glucose by -3.06 (95% CI, -5.77 to -0.36) mg/dL (test for heterogeneity and overall effect: $I^{2}=45.1\%$, p<0.05; z=2.20, p=0.028) per one copy of the *TCF7L2* T risk allele. Furthermore, regardless of genetic risk, diet/lifestyle interventions were associated with lower waist circumference. However, there was no significant change for diet/lifestyle interventions in other glycemic control and adiposity traits per one copy of *TCF7L2* risk allele.*Conclusions*. Our findings suggest that carrying the *TCF7L2* T risk allele may have a modestly greater benefit for specific diet/lifestyle interventions to improve the control of fasting glucose in overweight or obese adults.

## 1. Introduction

Type 2 diabetes (T2D) is a rapidly growing public health issue with a major effect on morbidity and mortality worldwide [1]. Both environmental and genetic factors have been implicated in the development of T2D [2– 4]. Recent genome wide association studies (GWAS) for T2D have identified more than 100 loci [5], whereof the single nucleotide polymorphism (SNP) within *TCF7L2* (transcription factor 7-like 2 gene), rs7903146, is the strongest and most widely replicated genetic marker for T2D across multiple populations [6, 7].

Although previous reports have shown that the *TCF7L2* variant was associated with glycemic control and weight changes [2, 8, 9], such genetic association may be modulated by dietary factors such as dietary fat [10], carbohydrate [3], and animal protein [11], but results from intervention studies are limited [2, 4, 9, 12, 13]. Both the Diabetes Prevention Program (DPP) and the Finnish Diabetes Prevention Study (DPS) reported that the diabetogenic effect of the *TCF7L2* variant rs7903146 was mitigated by lifestyle intervention [2, 12]. Likewise, the Mediterranean diet also modulates the effects of the *TCF7L2* rs7903146 on fasting glucose [13]. In contrast, in a population at risk for T2D, *TCF7L2* rs7903146 did not influence lifestyle intervention-induced changes in blood glucose, insulin secretion, and insulin sensitivity [9] or weight changes [4, 14]. Therefore, the association of *TCF7L2* with glycemic control in response to diet/lifestyle intervention is still controversial.

We carried out a large collaborative study of individual participant data to investigate whether the well-recognized *TCF7L2* variant rs7903146 associates with the changes in diabetes and obesity related outcomes in seven randomized lifestyle interventions with up to 4,114 overweight or obese participants enrolled. In particular, we investigated whether a randomized diet/lifestyle intervention modulates the genetic association of *TCF7L2* genotypes with glycemic control and adiposity changes.

## 2. Subjects and Methods

### 2.1. Study Participants

The study was conducted from October 2016 to May 2018 within the Gene-Lifestyle Intervention working Group (GIG), represented here by 7 randomized controlled trials (RCTs) with up to 4,114 individuals (eFigure 1 and eTable 1). This study followed the PRISMA reporting guideline for meta-analyses. Before collecting individual participant data, we searched systematically for studies published from inception to October 2016 through PubMed and Embase to identify intervention studies reporting weight loss, glycemic traits, or lipids by *TCF7L2* rs7903146 after a dietary or physical activity based intervention. Included studies were randomized intervention studies in overweight or obese participants aged more than 18 years. Only publications with an English language abstract were included. Studies in healthy participants and children (<18 years) were excluded. Two review authors independently evaluated overall study quality utilizing the standardized criteria (Grades of Recommendation, Assessment, Development and Evaluation Working Group (GRADE)). The detailed information of included RCTs were shown in eTable 1-3. Finally, 11 studies were eligible for inclusion, and 7 studies agreed to participate. A common analytical plan was used for all studies, with similar covariates included across studies as closely as possible. Individual participant data were first analyzed separately by each study, and then the results were pooled together using a random-effect meta-analysis [15]. Descriptions of each participating study (study design, study start data, study completion date, etc.) are shown in eTable 2. Participants from all participating trials provided written, informed consent, and ethical approval was granted by local institutional review boards. 

### 2.2. Diet/Lifestyle Interventions

We included seven randomized diet/lifestyle intervention trials among adults where the *TCF7L2* rs7903146 variant and outcomes (obesity measures or glycemic traits) were available (Table 1). Detailed information on study design and study-specific data collection methods are provided in eTable 2. Study information including study design, intervention type (dietary or lifestyle intervention), length of follow-up, country, participant characteristics (age, sex, and ethnicity), and description of measurement methods was also collected (Table 1 and eTables 1 and 3). 

**Table 1:** Characteristics of studies included (n=4,114).

Study	DIETFITS	Diogenes	FinDPS	NUGENOB	POUNDS Lost	PREDIMED	WAHA
No. of participants	435	634	344	517	734	824	626
Intervention type	Diet	Diet	Diet and exercise	Diet	Diet	Diet	Diet
Intervention length (weeks)	52	26	144	10	96	48	96
Region/ethnicity	North America ^$^	Europe	Europe	Europe	North America ^∗^	Europe	Europe/North America
Age (years)	42.9 (6.6)	55.1 (7.2)	54.8 (7.2)	36.8 (7.9)	51.6 (9.1)	67.6 (6.0)	69.5 (3.7)
BMI (kg/m ^2^)	33.0 (3.0)	34.3 (3.2)	31.1 (4.6)	35.7 (5.0)	32.6 (3.9)	29.2 (3.3)	27.2 (4.3)
Current smoking, %	0	26.4	NA	28	4	12.1	2.8
Active physical activity habit, %	77	31.2	79.1	59.3	100	40.8	50
HWE *p* value ^#^	<0.05	0.67	0.6	0.79	0.7	0.88	0.61

Values for age and BMI are expressed as mean (SD). BMI: body mass index; NA: not applicable. SNP: single nucleotide polymorphism; HWE: Hardy-Weinberg equilibrium. ^$^59% white, 4% black, 21% Hispanic, 10% Asian, and 6% other. ^∗^White (79%), black (16%), Hispanic (4%), and other (1%). ^#^P value for Hardy-Weinberg equilibrium test for *TFC7L2* SNP (rs7903146). Hardy-Weinberg equilibrium was tested by using the chi-square test.

### 2.3. Genetic Variant Selection and Genotype Properties

All studies used direct genotype information on rs7903146 from previously genotyped array data. Genotyping platforms, genotype frequencies, Hardy Weinberg equilibrium P values, and call rates (median of 98.8%) for *TCF7L2* rs7903146 were listed in eTable 4. 

### 2.4. Quantitative Measurements

Anthropometric measures and fasting blood samples were collected at baseline and endpoint of trial according to standard protocols. We used glucose and insulin measured in conventional units (milligrams per deciliter and microunits per milliliter, respectively) unless otherwise specified. The HOMA index was calculated as $\left(\text{fasting plasma insulin concentration}\left(\text{mU}/\text{L}\right)\times \text{fasting plasma glucose}\left(\text{mmol}/\text{L}\right)\right)/22.5$ for IR and $\text{HOMA}-\%\text{B}=\left(20\times \text{fasting plasma insulin concentration}\left(\text{mU}/\text{L}\right)\right)/\left(\text{fasting plasma glucose}\left(\text{mmol}/\text{L}\right)-3.5\right)$ for *β*-cell function, respectively. Conversion factors (1mg/dL=0.055mmol/L and 1μIU/mL=6.94pmol/L) for fasting glucose and fasting insulin were used to convert the conventional units to System International (SI) units. Detailed information on the outcome measures for each study is reported in eTable 3. 

### 2.5. Statistical Analyses

Statistical analyses were conducted using Stata 14.0 software (Stata, College Station, TX). Outcomes of interest were changes (calculated as follow-up measurement minus baseline measurement) in fasting glucose, fasting insulin, HOMA-IR, HOMA-B, body weight, and waist circumference versus control. Linear regression analyses were used to test the main effect of the *TCF7L2* genetic variant on changes in outcomes versus the control group. The *TCF7L2* genotype was coded using an allele-dose model (“CC” coded as “0,” “CT” coded as “1,” and “TT” coded as “2”). Interactions between the effect of the intervention group (versus control) and genotypes were tested by including an interaction term in the models as independent predictors of each outcome. We adjusted for age (continuous, year), sex, ethnicity (categorical), current smoking (yes or no), physical activity (active or inactive), body mass index (BMI, continuous, kg/m ^2^) at the baseline, each outcome trait at the baseline, and diet/intervention groups (eTable 5). Participants were excluded from the analyses if they did not have complete data for all outcomes and covariates. 

Two sets of regression coefficients are presented. The first arm-specific set of coefficients captures within-arm changes in the outcomes (from baseline to endpoint) for each copy of the *TCF7L2* risk allele. For studies with more than one active treatment arm, we combined all active treatment arms together, thus creating a single intervention versus control comparison for each study. For those trials where all arms received interventions, we define the low-fat or low-protein groups as control (e.g., the POUND Lost trial and NUGENOB). The second set of coefficients captures mean differences in *TCF7L2* allelic effects between treatment and control arms, where a negative coefficient means that individuals carrying a risk allele had a greater reduction in the outcomes in response to the interventions (versus control) than those without the risk allele. Importantly, the first set of coefficients, which was used for standard error calculations for the second set of coefficients that capture the gene treatment interaction [16], is estimated from separate arms. 

Heterogeneity between studies was evaluated using the $I^{2}$ test and Galbraith plot s [17] (eFigure 2). Random effects models were used to pool effect sizes and to account for both sampling error and between study variation in population [18]. Small study effects were assessed by visual inspection of funnel plots of effect size against the standard error [19], where a P value less than 0.1 was considered as significant [20] (eFigure 3). 

To explore potential sources of heterogeneity, we conducted moderation testing (subgroup analyses) using number of participants (<500 and ≥500), intervention type (diet and diet and/or exercise), intervention length (<48 weeks and ≥48 weeks), and age (<50 years and ≥ 50 years) as putative categorical moderators (eTable 6). We run a further sensitivity analysis by excluding the FinDPS (the only study that included both diet and lifestyle-based interventions) or by excluding the DIETFITS that did not achieve Hardy-Weinberg equilibrium (HWE). 

## 3. Results

### 3.1. Baseline Characteristics of Participating Studies

Characteristics of the 4,114 participants from 7 trials are shown in Table 1 and eTable 1. The mean age at baseline was 51.6 (ranged from 28 to 74) years, and the mean BMI was 32.2 (ranged from 23.8 to 43.2) kg/m ^2^. The frequency for the *TCF7L2* rs7903146 risk allele T ranges from 22% to 39% (eTable 4). Chi-square tests showed that the DIETFITS study did not achieve HWE (Table 1). Six studies were dietary interventions, and one is dietary and exercise-based intervention. The duration of intervention ranged from ten weeks to three years. All studies were conducted in North America and Europe; two study (POUNDS Lost and WAHA) were mixed in ethnicity (Table 1). 

### 3.2. Main Association of *TCF7L2* Genotype with Glycemic and Obesity Traits at Baseline or their Changes after Intervention 

At baseline, each copy of the *TCF7L2* T risk allele was marginally associated with a 0.118 mmol/L higher fasting glucose (95% CI, -0.017 to 0.254; p=0.057), but a 0.575 kg lower body weight (95% CI, -1.137 to -0.013; p=0.041). We did not observe significant associations for baseline waist circumference, fasting insulin, HOMA-IR, and HOMA-B (eFigure 3). 

Among all diet/lifestyle intervention groups (excluding control group), for each copy of the *TCF7L2* T allele, a lower level of waist circumference and a marginal lower body weight were observed (regression coefficients and standard error: −0.36±0.14, p=0.011; −0.20±0.11, p=0.061, respectively) (eTable 7). 

### 3.3. Gene Treatment Interaction on Glycemic Control and Adiposity Changes

**Figure 1:**
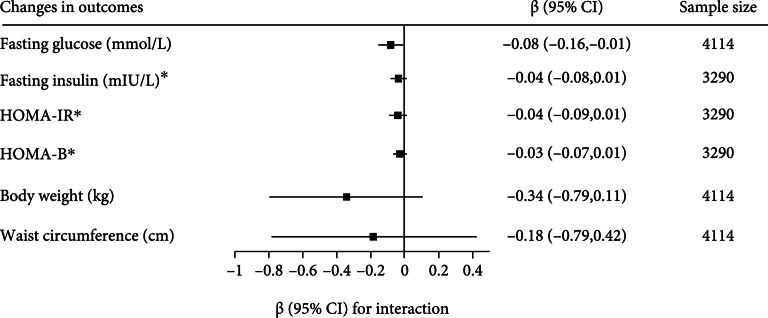
Gene treatment interaction for changes in outcomes. Values represent coefficient and 95% confidence interval for interactions between *TCF7L2* (rs7903146) treatments on changes in outcomes after intervention (allele dose model was employed and coded in terms of copies of minor allele (0, 1, 2)) in random effects meta-analysis of 4,114 adults. ^∗^Data on insulin, HOMA-IR, and HOMA-B are log transformed before analysis. The HOMA was calculated as $\left(\text{fasting plasma insulin concentration}\left(\text{mIU}/\text{L}\right)\times \text{fasting plasma glucose}\left(\text{mmol}/\text{L}\right)\right)/22.5$ for IR and $\text{HOMA}-\%\text{B}=\left(20\times \text{fasting plasma insulin concentration}\left(\text{mU}/\text{L}\right)\right)/\left(\text{fasting plasma glucose}\left(\text{mmol}/\text{L}\right)-3.5\right)$ for *β*-cell function. Fasting glucose was as follows: 1mg/dL=0.055mmol/L; fasting insulin was as follows: 1μIU/mL=6.94pmol/L.

After adjustment for age, sex, ethnicity, current smoking habit, physical activity level, BMI and each outcome-trait at baseline, significant interaction with diet/lifestyle treatment was observed for rs7903146 for changes in fasting glucose (p=0.023) (Figure 1). The interactions for change in fasting insulin, HOMA-IR, HOMA-B, body weight, and waist circumference were marginally significant, but they suggested a similar direction as the interaction for change in fasting glucose (Figure 1). 

**Figure 2:**
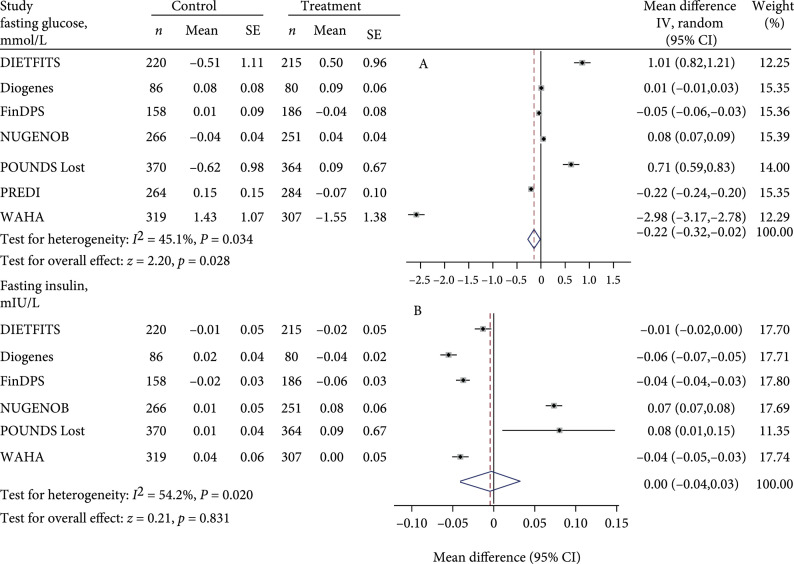
Forest plot of change in fasting glucose and insulin after intervention for each copy of the *TCF7L2* rs7903146 minor allele (T) in treatment versus control arm in random effects meta-analysis of 3,655 adults. Values for treatment and control represent coefficient and standard error from linear regression analyses adjusted for age, sex, baseline outcome, and ethnicity where appropriate. When more than one treatment arm was present, values represent combined effects across treatment arms. The gray areas around data points indicate the effect size of each study. The dotted line indicates the overall effect size. POUNDS Lost: The Preventing Overweight Using Novel Dietary Strategies trial; DIETFITS: Diet Intervention Examining the Factors Interacting with Treatment Success; DIOGENES: Diet, Obesity, and Genes; WAHA: Walnuts and Healthy Aging; FinDPS or FDPS: The Finnish Diabetes Prevention Trial; PREDIMED-Reus: Prevencion con dieta mediterranea-Reus; NUGENOB: Nutrient-Gene Interactions in Human Obesity; IV: Inverse Variance. Fasting glucose: 1mg/dL=0.055mmol/L; fasting insulin: 1μIU/mL=6.94pmol/L.

**Figure 3:**
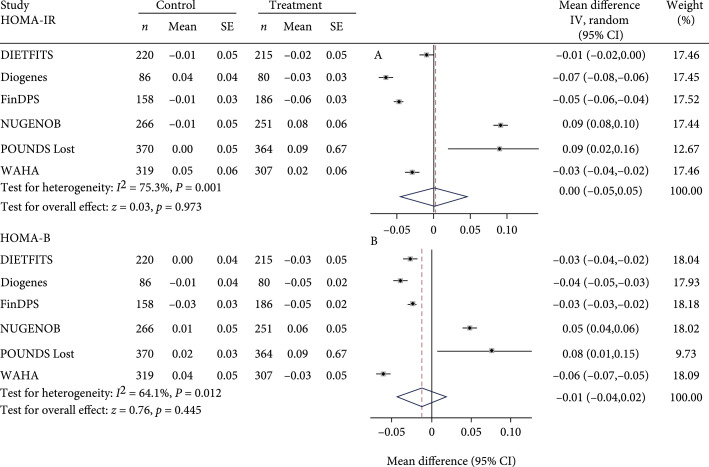
Forest plot of change in HOMA-IR and HOMA-B after intervention for each copy of the *TCF7L2* rs7903146 minor allele (T) in treatment versus control arm in random effects meta-analysis of 3,655 adults. Values for treatment and control represent coefficient and standard error from linear regression analyses adjusted for age, sex, baseline outcome, and ethnicity where appropriate. When more than one treatment arm was present, values represent combined effects across treatment arms.

**Figure 4:**
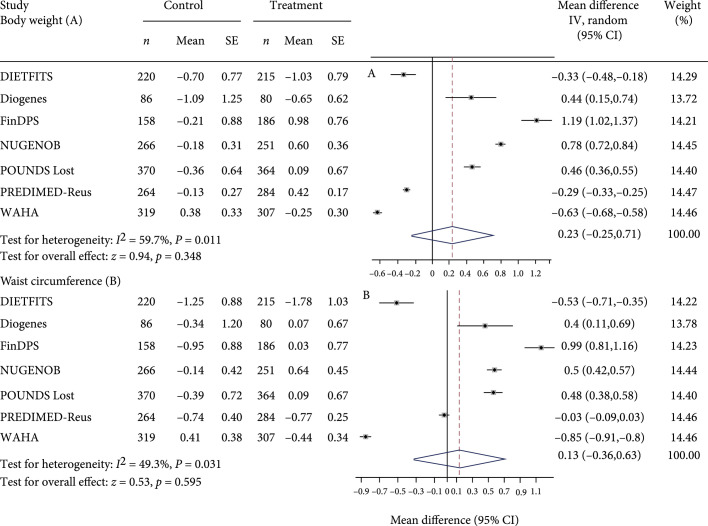
Forest plot of change in body weight and waist circumference after intervention for each copy of the *TCF7L2* rs7903146 minor allele (T) in treatment versus control arm in random effects meta-analysis of 3,655 adults. Values for treatment and control represent coefficient and standard error from linear regression analyses adjusted for age, sex, baseline outcome, and ethnicity where appropriate. When more than one treatment arm was present, values represent combined effects across treatment arms.

Figure 2 summarizes the risk allele effects for each study or arm and differences in risk allele effects between treatment and control arms on glycemic and obesity outcomes after the intervention. Mean treatment versus control differences in fasting glucose change for each copy of the *TCF7L2* T allele ranged from -53.69 mg/dL with 95% CI (-57.12, -50.09) in WAHA to 18.20 mg/dL with 95% CI (14.77, 21.80) in the DIETFITS study. Compared with control, diet/lifestyle treatment associated with lower fasting glucose level by -3.06 (95% CI, -5.77 to -0.36) mg/dL (test for heterogeneity: $I^{2}=45.1\%$, p<0.05; test for overall effect: z=2.20, p=0.028) for each copy of the *TCF7L2* T allele. When subjects received a diet/lifestyle treatment, individuals with the T allele of rs7903146 had a lower fasting glucose level than did carriers of the non-T allele. We did not observe significant difference in risk allele effects between treatment and control arms for fasting insulin (Figure 2), HOMA-IR, HOMA-B (Figure 3), body weight, and waist circumference (Figure 4). 

### 3.4. Stratified and Sensitivity Analyses

The associations between *TCF7L2* genotype and differences between treatment and control in the change in fasting glucose after the diet/lifestyle interventions were influenced by number of participants, study length, and age, but not by study type (eTable 8). Mean treatment versus control differences in fasting glucose change for each copy of the *TCF7L2* T allele was significant in participants aged 50 or older (-0.45 (-0.67, -0.23); p<0.001), but not in those younger than 50 years (0.54 (-0.37, 1.45); p=0.247). We conducted further sensitive analyses by excluding the FinDPS, the only study that used diet and excise as treatment, and the DIETFITS study that did not achieve HWE. This did not change the pattern of results (data not shown). Likewise, after removing NUGENOB, the shortest study (10 weeks), overall effect sizes for fasting glucose did not change. 

## 4. Discussion

The present collaborative study used individual participant data to investigate if diet/lifestyle interventions modify the association of *TCF7L2* genotypes with glycemic control and adiposity in RCTs. Our analysis of seven studies involving 4,114 adults showed that carrying the *TCF7L2* rs7903146 T risk allele was significantly associated with changes in fasting glucose in response to diet/lifestyle treatment, compared with non-T allele carriers. Specifically, people who carried the *TCF7L2* T allele responded better to the intervention in terms of glycemic control. Furthermore, our findings suggest that diet/lifestyle interventions associated with lower waist circumference regardless of genetic risk.

The *TCF7L2* is the strongest common locus associated with T2D identified thus far [6]. *TCF7L2* variants have been shown to associate with impaired glucose tolerance and insulin synthesis and secretion and to predict the incidence of T2D in persons who were already at high risk of the disease [2], which suggests potential synergistic effects between different risk factors. Two large trials (DPP and DPS) showed that the diabetogenic effect of the variant rs7903146 in *TCF7L2* was mitigated by lifestyle intervention [2, 12]; although, a small study (n=309) of lifestyle intervention in a population at risk for T2D did not observe such interaction on changes in blood glucose, insulin secretion, or insulin sensitivity [9]. In the present study, we found modulation by the diet/lifestyle intervention on changes in fasting glucose, suggesting that diet/lifestyle treatment results in a greater decrease in fasting glucose in TT individuals compared with controls. Although the interaction effects on fasting insulin and insulin resistance were not statistically significant, the directions of the changes were consistent with those of fasting glucose. We speculate that the medications used by participants that might interfere with HOMA assessments by modifying insulin secretion and sensitivity might explain the negative association. Our findings imply that the genetic predisposition to diabetes from the *TCF7L2* risk allele can be at least partly counteracted by diet or exercise-based intervention and that those carrying the risk allele respond well to such interventions.

The mechanisms underlying our findings are unknown but may be related to the potential role of *TCF7L2* in regulating hepatic glucose metabolism. *TCF7L2* is a Wnt signaling-associated transcription factor expressed in several tissues, including the liver and pancreas [21]. Previous evidence showed that the *TCF7L2* risk alleles might act through gene-expression, with the mRNA expression levels of the *TCF7L2* being almost 3-fold higher for individuals with the rs7903146 T risk allele compared to those with non-T alleles [22]. In addition, rodent studies and cell culture experiments demonstrated that high-fat diet feeding reduced the expression of the *TCF7L2* gene in the pancreas [23]. Therefore, diet/lifestyle interventions may differently influence the expression of the *TCF7L2* gene and regulate fasting glucose levels in the context of genotypes.

In the current large collaborative analysis, we observed that the *TCF7L2* T allele was associated with greater reduced waist circumference among diet/lifestyle treatment group, but the *TCF7L2* genotype was not an effect modifier. Nonetheless, our findings are in line with the results of the large DPP trial, which also found no interaction between genotype and a lifestyle intervention on body weight changes [14] and suggested that *TCF7L2* may not determine the ability to lose weight. Similarly, the POUNDS Lost trial observed no interaction for changes in weight, waist circumference, or body fat when assessed at 24 months [4]. In contrast, previous findings in a population with impaired glucose tolerance showed that the lifestyle intervention induced changes in BMI, total fat, and nonvisceral and visceral fat that varied according to *TCF7L2* rs7903146 genotypes [9]. Differences in dietary composition, time frame, other lifestyle changes, and populations may have accounted for discrepancies among studies. Although body composition was outside the scope of the present analysis, there is good evidence that *TCF7L2* rs7903146 might modulate the influence of diet/lifestyle on lean mass [4], fat-free mass and fat mass [24], total body fat, and visceral and nonvisceral fat [9]. It is important to elucidate the potential mechanisms through which *TCF7L2* rs7903146 influences body composition in further research.

The major strength of the present large collaborative study is that we tested gene-diet/lifestyle interaction under RCT settings, which avoids residual confounding biases characteristic of observational study designs. Moreover, our study comprising 4,114 adults from seven dietary intervention trials provided adequate statistical power for our analyses. In addition, the use of standardized statistical analyses improved the overall reliability of the results by allowing adjustment for the same set of covariates across all studies.

An important limitation is that we evaluated a single genetic variant effect of *TCF7L2* genotype. Although previous meta-analysis of RCTs demonstrated that the *FTO* genotype did not influence the change in adiposity after weight loss interventions [16], it is worth noting that both diabetes and obesity risks are modified by multiple genetic variants; therefore, our findings for *TCF7L2* genotype should not be considered in isolation. Nonetheless, our findings are in line with those from the DPP and Finnish DPS trials [2, 12] and the PREDIMED trial [13], whereby participants benefited from diet/lifestyle modification regardless of genetic risk. Second, studies included in our analysis varied in type of interventions investigated, study design, duration, sample size, ethnicity, and health condition, and such heterogeneity might influence our ability to identify an interaction effect. We acknowledge that the controls of some studies such as the POUNDS Lost trial, NUGENOB, and DIETFITS study also received treatments, making the interpretation of our results difficulty. Third, we observed that the *TCF7L2* T allele was associated with a lower level of waist circumference and a marginal lower body weight after diet/lifestyle intervention. These results should be interpreted cautiously. When body weight and waist circumference are evaluated in cohorts ascertained for glycemic status, collider bias might be introduced, producing an artificial association of the diabetes-raising allele with lower body weight or waist circumference. It is worth noting that the selected participants from the seven included studies were overweight or obese; therefore, the *TCF7L2* variant and adiposity association analyses might be prone to a particular bias inducing paradoxical results, namely, index event bias. Fourth, we did not consider details on how smoking was assessed, including the frequency, duration, and number of cigarettes. In addition, accurate measurements of physical activity (e.g,. MET) are also needed in further study. Fifth, we did not take the effect of drug use into consideration. Sixth, since participants with missing information on outcomes and covariates could not be analyzed, we did not calculate the number of those participants and whether there were significant differences between those who were included and those who were not, which may result in biased results to some extent. Seventh, we did not perform stratified meta-analyses by sex which may be warranted in future study. Eighth, studies included in this meta-analysis could not represent all types of diet/lifestyle interventions, which limit the generalizability of the findings to other types of diet/lifestyle interventions. Finally, all trials analyzed here were conducted in North America or Europe, with predominantly white participants, which limit the generalizability of our findings to other ethnicities. Given evidence that the genetic effect of the *TCF7L2* genotype varies across ethnicities, further studies on this topic in different ethnic populations are warranted.

In summary, this large collaborative analysis of individual participant data suggests that carrying the *TCF7L2* risk allele may confer modestly greater benefit in improving glycemic control in response to specific diet/lifestyle interventions in overweight or obese adults. Understanding the mechanisms by which variation in this gene affects glucose homeostasis may provide new insights into the molecular basis of diabetes and opportunities for targeted preventive interventions.

## Data Availability

Raw data is not available, while summary data is available on request (Dr. Tao Huang, huangtao@bjmu.edu.cn).

## Supplementary Materials

Supplementary Materials Supplemental Figure 1: participant flow chart. Supplemental Figure 2: Galbraith plots to estimate small-study effects of studies (n=4114) for changes in fasting glucose, insulin, HOMA-IR, HOMA-B, body weight, and waist circumference. Supplemental Figure 3: forest plot of mean differences in baseline fasting glucose, insulin, HOMA-IR, HOMA-B, body weight, and waist circumference for each copy of the TCF7L2 rs7903146 risk allele (T). Supplemental Table 1: population characteristics of participants. Supplemental Table 2: descriptions of studies. Supplemental Table 3: outcome measurements. Supplemental Table 4: genotyping information in included studies. Supplemental Table 5: covariates included in nonstratified linear regression models. Supplemental Table 6: characteristics of studies included in qualitative synthesis (n=4114). Supplemental Table 7: main genetic effect of TCF7L2 rs7903146 on changes in outcomes among all intervention groups (excluding control group). Supplemental Table 8: stratified analyses to identify potential moderators of relation between TFC7L2 rs7903146 genotype and intervention effects on change in outcomes.

## References

[1] P. Zimmet, K. G. Alberti, D. J. Magliano, and P. H. Bennett, “Diabetes mellitus statistics on prevalence and mortality: facts and fallacies,” *Nature Reviews. Endocrinology*, vol. 12, no. 10, pp. 616–622, 2016 10.1038/nrendo.2016.105 27388988

[2] J. C. Florez, K. A. Jablonski, N. Bayley, T. I. Pollin, P. I. de Bakker, A. R. Shuldiner, W. C. Knowler, D. M. Nathan, D. Altshuler, and Diabetes Prevention Program Research Group, “TCF7L2 polymorphisms and progression to diabetes in the diabetes prevention program,” *The New England Journal of Medicine*, vol. 355, no. 3, pp. 241–250, 2006 16855264 10.1056/NEJMoa062418 PMC1762036

[3] M. C. Cornelis, L. Qi, P. Kraft, and F. B. Hu, “TCF7L2, dietary carbohydrate, and risk of type 2 diabetes in US women,” *The American Journal of Clinical Nutrition*, vol. 89, no. 4, pp. 1256–1262, 2009 19211816 10.3945/ajcn.2008.27058 PMC2667467

[4] J. Mattei, Q. Qi, F. B. Hu, F. M. Sacks, and L. Qi, “TCF7L2 genetic variants modulate the effect of dietary fat intake on changes in body composition during a weight-loss intervention,” *The American Journal of Clinical Nutrition*, vol. 96, no. 5, pp. 1129–1136, 2012 23034957 10.3945/ajcn.112.038125 PMC3471200

[5] eQTLGen Consortium, A. Xue, Y. Wu, Z. Zhu, F. Zhang, K. E. Kemper, Z. Zheng, L. Yengo, L. R. Lloyd-Jones, J. Sidorenko, Y. Wu, A. F. McRae, P. M. Visscher, J. Zeng, and J. Yang, “Genome-wide association analyses identify 143 risk variants and putative regulatory mechanisms for type 2 diabetes,” *Nature Communications*, vol. 9, no. 1, p. 2941, 2018 10.1038/s41467-018-04951-w PMC6063971 30054458

[6] S. F. Grant, G. Thorleifsson, I. Reynisdottir, R. Benediktsson, A. Manolescu, J. Sainz, A. Helgason, H. Stefansson, V. Emilsson, A. Helgadottir, U. Styrkarsdottir, K. P. Magnusson, G. B. Walters, E. Palsdottir, T. Jonsdottir, T. Gudmundsdottir, A. Gylfason, J. Saemundsdottir, R. L. Wilensky, M. P. Reilly, D. J. Rader, Y. Bagger, C. Christiansen, V. Gudnason, G. Sigurdsson, U. Thorsteinsdottir, J. R. Gulcher, A. Kong, and K. Stefansson, “Variant of transcription factor 7-like 2 (TCF7L2) gene confers risk of type 2 diabetes,” *Nature Genetics*, vol. 38, no. 3, pp. 320–323, 2006 16415884 10.1038/ng1732

[7] T. Jin, “Current understanding on role of the Wnt signaling pathway effector TCF7L2 in glucose homeostasis,” *Endocrine Reviews*, vol. 37, no. 3, pp. 254–277, 2016 27159876 10.1210/er.2015-1146

[8] S. Cauchi, H. Choquet, R. Gutiérrez-Aguilar, F. Capel, K. Grau, C. Proença, C. Dina, A. Duval, B. Balkau, M. Marre, N. Potoczna, D. Langin, F. Horber, T. I. Sørensen, G. Charpentier, D. Meyre, and P. Froguel, “Effects of TCF7L2 polymorphisms on obesity in European populations,” *Obesity (Silver Spring)*, vol. 16, no. 2, pp. 476–482, 2008 18239663 10.1038/oby.2007.77

[9] A. Haupt, C. Thamer, M. Heni, C. Ketterer, J. Machann, F. Schick, F. Machicao, N. Stefan, C. D. Claussen, H. U. Haring, A. Fritsche, and H. Staiger, “Gene variants of TCF7L2 influence weight loss and body composition during lifestyle intervention in a population at risk for type 2 diabetes,” *Diabetes*, vol. 59, no. 3, pp. 747–750, 2010 20028944 10.2337/db09-1050 PMC2828665

[10] C. M. Phillips, L. Goumidi, S. Bertrais, M. R. Field, R. McManus, S. Hercberg, D. Lairon, R. Planells, and H. M. Roche, “Dietary saturated fat, gender and genetic variation at the _TCF7L2_ locus predict the development of metabolic syndrome,” *The Journal of Nutritional Biochemistry*, vol. 23, no. 3, pp. 239–244, 2012 21543200 10.1016/j.jnutbio.2010.11.020

[11] E. Fisher, K. Meidtner, L. Ängquist, C. Holst, R. D. Hansen, J. Halkjœr, G. Masala, J. N. Østergaard, K. Overvad, D. Palli, K. S. Vimaleswaran, A. Tjønneland, D. L. van der A, N. J. Wareham, T. I. A. Sørensen, R. J. F. Loos, and H. Boeing, “Influence of dietary protein intake and glycemic index on the association between TCF7L2 HapA and weight gain,” *American Journal of Clinical Nutrition*, vol. 95, no. 6, pp. 1468–1476, 2012 22552033 10.3945/ajcn.111.014670

[12] J. Wang, J. Kuusisto, M. Vänttinen, T. Kuulasmaa, J. Lindström, J. Tuomilehto, M. Uusitupa, and M. Laakso, “Variants of transcription factor 7-like 2 (TCF7L2) gene predict conversion to type 2 diabetes in the Finnish Diabetes Prevention Study and are associated with impaired glucose regulation and impaired insulin secretion,” *Diabetologia*, vol. 50, no. 6, pp. 1192–1200, 2007 17437080 10.1007/s00125-007-0656-6

[13] D. Corella, P. Carrasco, J. V. Sorli, R. Estruch, J. Rico-Sanz, M. A. Martinez-Gonzalez, J. Salas-Salvado, M. I. Covas, O. Coltell, F. Aros, J. Lapetra, L. Serra-Majem, V. Ruiz-Gutierrez, J. Warnberg, M. Fiol, X. Pinto, C. Ortega-Azorin, M. A. Munoz, J. A. Martinez, E. Gomez-Gracia, J. I. Gonzalez, E. Ros, and J. M. Ordovas, “Mediterranean diet reduces the adverse effect of the TCF7L2-rs7903146 polymorphism on cardiovascular risk factors and stroke incidence: a randomized controlled trial in a high-cardiovascular-risk population,” *Diabetes Care*, vol. 36, no. 11, pp. 3803–3811, 2013 23942586 10.2337/dc13-0955 PMC3816851

[14] J. M. McCaffery, K. A. Jablonski, P. W. Franks, S. Dagogo-Jack, R. R. Wing, W. C. Knowler, L. Delahanty, D. Dabelea, R. Hamman, A. R. Shuldiner, J. C. Florez, and for the Diabetes Prevention Program Research Group, “TCF7L2 polymorphism, weight loss and proinsulin: insulin ratio in the Diabetes Prevention Program,” *PLoS One*, vol. 6, no. 7, p. e21518, 2011 21814547 10.1371/journal.pone.0021518 PMC3144193

[15] R. D. Riley, P. C. Lambert, and G. Abo-Zaid, “Meta-analysis of individual participant data: rationale, conduct, and reporting,” *BMJ*, vol. 340, no. feb05 1, p. c221, 2010 20139215 10.1136/bmj.c221

[16] K. M. Livingstone, C. Celis-Morales, G. D. Papandonatos, B. Erar, J. C. Florez, K. A. Jablonski, C. Razquin, A. Marti, Y. Heianza, T. Huang, F. M. Sacks, M. Svendstrup, X. Sui, T. S. Church, T. Jääskeläinen, J. Lindström, J. Tuomilehto, M. Uusitupa, T. Rankinen, W. H. M. Saris, T. Hansen, O. Pedersen, A. Astrup, T. I. A. Sørensen, L. Qi, G. A. Bray, M. A. Martinez-Gonzalez, J. A. Martinez, P. W. Franks, J. M. McCaffery, J. Lara, and J. C. Mathers, “FTO genotype and weight loss: systematic review and meta-analysis of 9563 individual participant data from eight randomised controlled trials,” *BMJ*, vol. 354, p. i4707, 2016 27650503 10.1136/bmj.i4707 PMC6168036

[17] J. P. Higgins, and S. G. Thompson, “Quantifying heterogeneity in a meta-analysis,” *Statistics in Medicine*, vol. 21, no. 11, pp. 1539–1558, 2002 12111919 10.1002/sim.1186

[18] M. Borenstein, L. V. Hedges, J. P. Higgins, and H. R. Rothstein, “A basic introduction to fixed-effect and random-effects models for meta-analysis,” *Research Synthesis Methods*, vol. 1, no. 2, pp. 97–111, 2010 26061376 10.1002/jrsm.12

[19] Y. Hayashino, Y. Noguchi, and T. Fukui, “Systematic evaluation and comparison of statistical tests for publication bias,” *Journal of Epidemiology*, vol. 15, no. 6, pp. 235–243, 2005 16276033 10.2188/jea.15.235 PMC7904376

[20] M. Egger, G. Davey Smith, M. Schneider, and C. Minder, “Bias in meta-analysis detected by a simple, graphical test,” *BMJ*, vol. 315, no. 7109, pp. 629–634, 1997 9310563 10.1136/bmj.315.7109.629 PMC2127453

[21] S. F. Boj, J. H. van Es, M. Huch, V. S. W. Li, A. José, P. Hatzis, M. Mokry, A. Haegebarth, M. van den Born, P. Chambon, P. Voshol, Y. Dor, E. Cuppen, C. Fillat, and H. Clevers, “Diabetes risk gene and Wnt effector _Tcf7l2_ /TCF4 controls hepatic response to perinatal and adult metabolic demand,” *Cell*, vol. 151, no. 7, pp. 1595–1607, 2012 23260145 10.1016/j.cell.2012.10.053

[22] D. X. Pang, A. J. P. Smith, and S. E. Humphries, “Functional analysis of _TCF7L2_ genetic variants associated with type 2 diabetes,” *Nutrition, Metabolism and Cardiovascular Diseases*, vol. 23, no. 6, pp. 550–556, 2013 10.1016/j.numecd.2011.12.012 PMC3778915 22402060

[23] J. Columbus, Y. T. Chiang, W. J. Shao, N. N. Zhang, D. Y. Wang, H. Y. Gaisano, Q. Wang, D. M. Irwin, and T. Jin, “Insulin treatment and high-fat diet feeding reduces the expression of three Tcf genes in rodent pancreas,” *The Journal of Endocrinology*, vol. 207, no. 1, pp. 77–86, 2010 20675304 10.1677/JOE-10-0044

[24] K. Grau, S. Cauchi, C. Holst, A. Astrup, J. A. Martinez, W. H. M. Saris, E. E. Blaak, J. M. Oppert, P. Arner, S. Rössner, I. A. Macdonald, E. Klimcakova, D. Langin, O. Pedersen, P. Froguel, and T. I. A. Sørensen, “TCF7L2 rs7903146-macronutrient interaction in obese individuals' responses to a 10-wk randomized hypoenergetic diet,” *American Journal of Clinical Nutrition*, vol. 91, no. 2, pp. 472–479, 2010 20032493 10.3945/ajcn.2009.27947

