# Supplementary material for: Interaction of Diet/Lifestyle Intervention and TCF7L2 Genotype on Glycemic Control and Adiposity among Overweight or Obese Adults: Big Data from Seven Randomized Controlled Trials Worldwide
Source: Health Data Sci. 2021 Nov 3;2021:9897048. doi: 10.34133/2021/9897048 (PMC10904069; doi:10.34133/2021/9897048)
Supplement: Supplementary Materials — Supplemental Figure 1: participant flow chart. Supplemental Figure 2: Galbraith plots to estimate small-study effects of studies (n=4114) for changes in fasting glucose, insulin, HOMA-IR, HOMA-B, body weight, and waist circumference. Supplemental Figure 3: forest plot of mean differences in baseline fasting glucose, insulin, HOMA-IR, HOMA-B, body weight, and waist circumference for each copy of the TCF7L2 rs7903146 risk allele (T). Supplemental Table 1: population characteristics of participants. Supplemental Table 2: descriptions of studies. Supplemental Table 3: outcome measurements. Supplemental Table 4: genotyping information in included studies. Supplemental Table 5: covariates included in nonstratified linear regression models. Supplemental Table 6: characteristics of studies included in qualitative synthesis (n=4114). Supplemental Table 7: main genetic effect of TCF7L2 rs7903146 on changes in outcomes among all intervention groups (excluding control group). Supplemental Table 8: stratified analyses to identify potential moderators of relation between TFC7L2 rs7903146 genotype and intervention effects on change in outcomes. [file 9897048.f1.docx]

**Supplemental Figure 1. Participant flow chart**

**Supplemental Figure 2. Galbraith plots to estimate small-study effects of studies (n=4114) for changes in fasting glucose, insulin, HOMA-IR, HOMA-B, body weight, and waist circumference.**

**Supplemental Figure 3. Forest plot of mean differences in baseline fasting glucose, insulin, HOMA-IR, HOMA-B, body weight, and waist circumference for each copy of the *TCF7L2* rs7903146 risk allele (T).**

**Supplemental Table 1. Population characteristics of participants**

**Supplemental Table 2. Descriptions of studies**

**Supplemental Table 3. Outcome measurements**

**Supplemental Table 4. Genotyping information in included studies.**

**Supplemental Table 5. Covariates included in non-stratified linear regression models**

**Supplemental Table 6. Characteristics of studies included in qualitative synthesis (n=4114).
Supplemental Table 7. Main genetic effect of *TCF7L2* rs7903146 on changes in outcomes among all intervention groups (excluding control group)**

**Supplemental Table 8. Stratified analyses to identify potential moderators of relation between *TFC7L2* rs7903146 genotype and intervention effects on change in outcomes**

**Supplemental Figure 1. Participant flow chart**

**
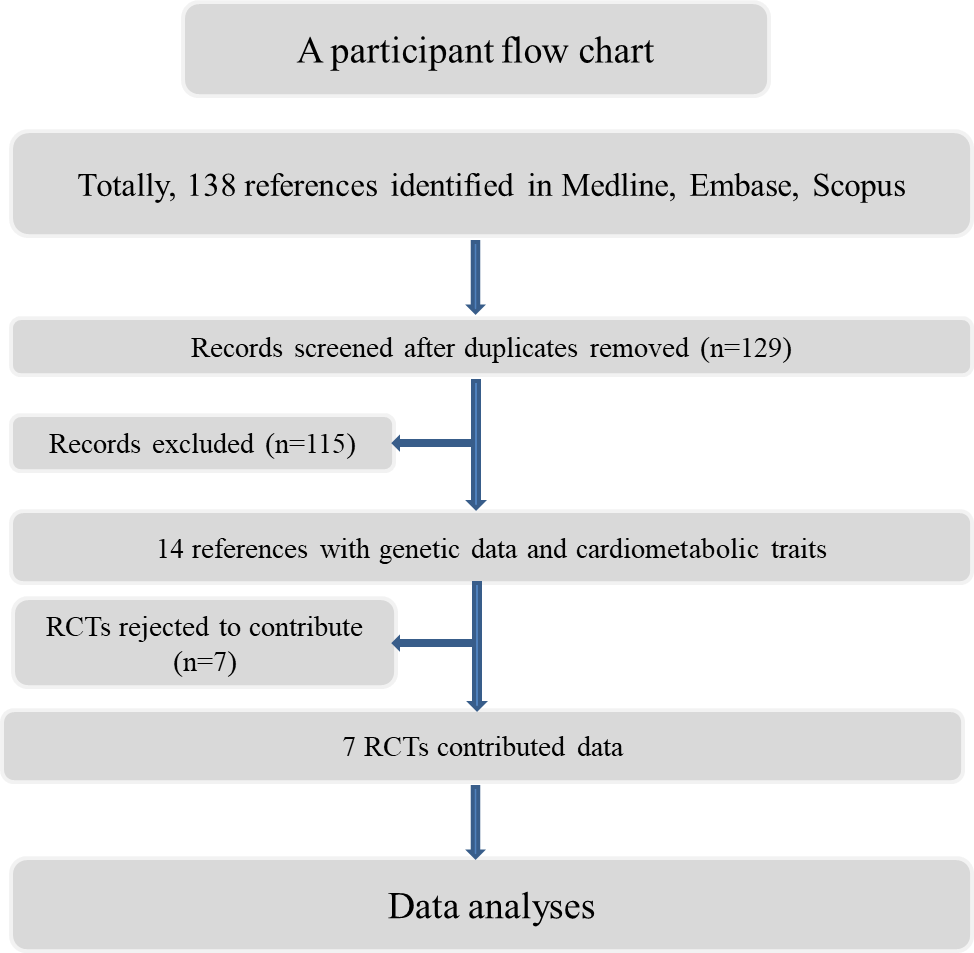
**

**Supplemental Figure 2. Galbraith plots to estimate small-study effects of studies (n=4114) for changes in fasting glucose, insulin, HOMA-IR, HOMA-B, body weight, and waist circumference.**

| **Glucose** | **Insulin** |
| --- | --- |
| **** | **** |
| **HOMA-IR** | **HOMA-B** |
| **** | **** |
| **Body weight** | **Waist circumference** |
| **** | **** |

Heterogeneity between studies was evaluated using the I^2^ test and Galbraith plots.

**Supplemental Figure 3 Forest plot of mean differences in baseline fasting glucose, insulin, HOMA-IR, HOMA-B, body weight, and waist circumference for each copy of the *TCF7L2* rs7903146 risk allele (T).**

**
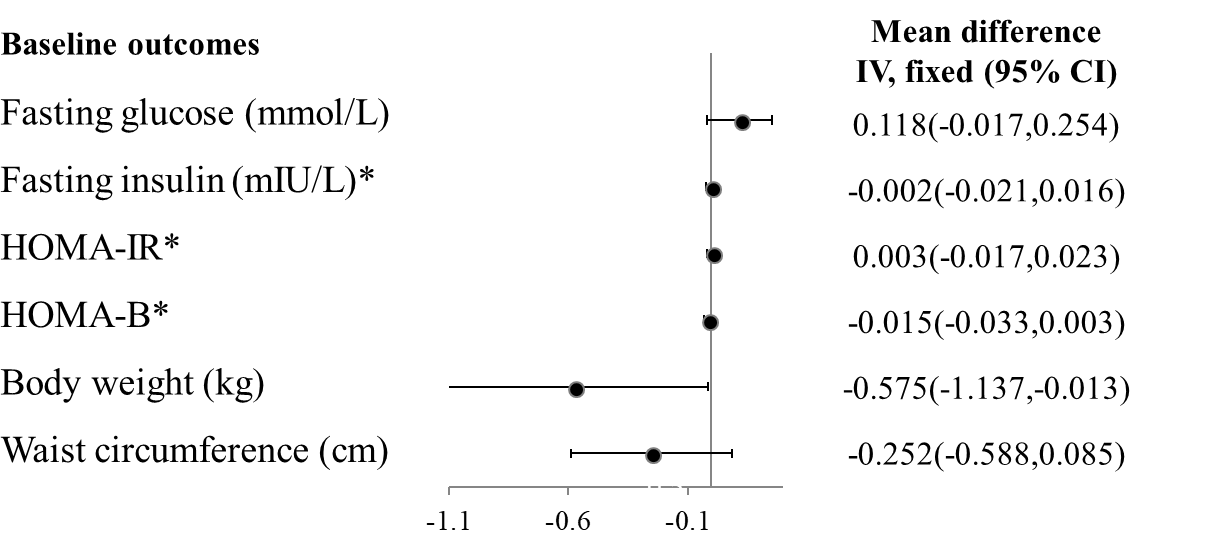
**

Values for treatment and control represent coefficient and 95% confidence interval from linear regression analyses adjusted for age, sex, baseline outcome, ethnicity where appropriate.

**Supplemental Table 1. Population characteristics of participants**

| **Study name** | | **Registration number** | **Blind (Unblinded, Single-blind, double-blind, triple-blind)** | **Randomization (yes, no)** | **Genotype** | **Software for analysis** |
| --- | --- | --- | --- | --- | --- | --- |
| **Full Name** | **Abbreviation** |  |  |  | **Genotyping method** | **SAS/R/SPSS/STATA** |
| The Preventing Overweight Using Novel Dietary Strategies trial | POUNDS Lost | ClinicalTrials.gov number, NCT00072995. | Double-blind | Yes | TaqMan | SAS |
| Diet Intervention Examining The Factors Interacting with Treatment Success | DIETFITS | NCT01826591 | Double-blind | Yes | Afflymetrix UK Biobank Axiom microarray | STATA |
| Diet, Obesity and Genes | DIOGENES | ClinicalTrials.gov number, NCT00390637 | Double-blind | yes | Illumina Exome SNP arrays. | SPSS, R |
| Walnuts and Healthy Aging | WAHA | ClinicalTrials.gov number NCT01634841 | Single- blind | Yes | TaqMan SNP specific assays | SPSS |
| The Finnish Diabetes Prevention Trial | FinDPS or FDPS | Clinical Trials.gov NCT00518167 | Lifestyle intervention | Yes | TaqMan | SAS |
| Prevencion con dieta mediterranea-Reus | PREDIMED-Reus | Clinical trial number: SRCTN35739639 ( www.controlled-trials.com ). | Unblinded | Yes | TaqMan | SPSS |
| Nutrient-Gene Interactions in Human Obesity | NUGENOB | ISRCTN25867281 (www.isrctn.com) | Open-label | Yes | Illumina MetaboChip. | R (version 3.2.5) |

**Supplemental Table 2. Descriptions of studies**

| POUNDS Lost is a randomized diet intervention trial in which 811 overweight and obese individuals were assigned to one of four energy-reduced diets varying in macronutrient composition of fat, protein, and carbohydrate to compare their effects on body weight change over 2 years. Two diets were low-fat (20%), and the other two diets were high-fat (40%), and two diets were average-protein (15%), and the other two diets were high-protein (25%), which constituted a 2-by-2 factorial design. The study was conducted from October 2004 through December 2007 at two sites: Harvard T.H. Chan School of Public Health and Brigham and Women’s Hospital in Boston, MA, and the Pennington Biomedical Research Center of Louisiana State University System, in Baton Rouge, LA. |
| --- |
| DIETFITS: This single-site, parallel-group, weight loss diet trial randomized individuals to a healthy low-fat diet or a healthy low-carbohydrate diet for 12 months. Participant enrollment began on January 29, 2013, and continued through April 14, 2015. The date of final follow-up was May 16, 2016. Interventions consisted primarily of class-based instruction. Five waves of recruitment (cohorts) had staggered start dates between March 2013 and March 2015. The primary outcome was 12-month weight change. The first primary hypothesis was that there is a significant diet × genotype pattern interaction for weight loss. The second primary hypothesis was that there is a significant diet × insulin secretion interaction for weight loss. Secondary outcomes included anthropometric measures, plasma lipid levels, insulin and glucose levels, and blood pressure levels. |
| DiOGenes is a pan-European controlled dietary intervention study (2006-2008) in 932 overweight adults who first lost body weight on an 8-week low-calorie diet and were then randomized to 1 of 5 ad libitum diets for 26 weeks. The diets were either high or low protein or high or low glycemic index in 4 combinations or control. At the 8 participating study centers (Maastricht, Netherlands; Copenhagen, Denmark; Cambridge, United Kingdom; Heraklion, Greece; Potsdam, Germany; Pamplona, Spain; Sofia, Bulgaria; and Prague, Czech Republic), the participating families received dietary instruction for a 26-week period. |
| WAHA is an RCT in which 708 cognitively healthy older individuals were assigned to habitual diet plus walnuts or habitual diet without nuts to compare effects on cognitive function and age-related macular degeneration over 2 years. Study conducted from May 2012 to May 2016 at two sites: Endocrinology & Nutrition Dept., Hospital Clínic of Barcelona, Spain and Center for Nutrition, Healthy Lifestyle and Disease Prevention, School of Public Health, Loma Linda University, Loma Linda, California. |
| The DPS was a randomized, controlled, multicenter study carried out in Finland between the years 1993 and 2000 (ClinicalTrials.govNCT00518167). A total of 522 individuals with impaired glucose tolerance (IGT) were randomized into either a lifestyle intervention or control group in five centres. The main inclusion criteria were as follows: BMI >25 kg/m2, age 40-64 years, and IGT based on the mean values of two OGTTs based on the World Health Organization 1985 criteria. Random allocation to one of the two study groups was stratified according to the centre, sex, and the 2-h glucose at the screening OGTT. At baseline and at annual visits, individuals completed a medical history questionnaire and underwent physical examination that included anthropometric measurements and an OGTT. The median length of the study was four years (range 1-6 years). During this four-year follow-up period all the participants were undergoing the randomized intervention. |
| The PREDIMED-Reus study is a randomized and controlled diet intervention trial. Eligible participants were community-dwelling persons (55-80 years for men; 60-80 years for women) who fulfilled at least one of two criteria: type2 diabetes or 3 or more cardiovascular risk factors. Patients with BMI > 45 were excluded. It was initiated in 2003 in the city of Reus, one of the sites of the multicenter PREDIMED trial. Here we included 824 participants with genotype data and analyzed changes after 1-year dietary intervention in three groups: Control group (advice to follow a low-fat diet), Mediterranean diet intervention group supplemented with extra virgin olive oil and Mediterranean diet intervention group supplemented with mixed nuts. |
| The NUGENOB study is a multi-center 10-week European diet intervention study investigating differences between two equally hypo-caloric diets and gene-diet interactions s at eight sites in seven European countries: United Kingdom (England), The Netherlands, France (two centers), Spain, Czech Republic, Sweden and Denmark. The study cohort consisted of 579 Caucasian women and 192 Caucasian men aged 20-50 years with obesity who were randomized to a low-fat/high-carbohydrate or a high-fat/low-carbohydrate diet. Recruitment of subjects was undertaken from May 2001 until September 2002. Inclusion and exclusion criteria, randomization procedure and blinding were previously described in details (ref: Petersen M, Taylor MA, Saris WH, Verdich C, Toubro S, Macdonald I, et al. Randomized, multi-center trial of two hypo-energetic diets in obese subjects: high- versus low-fat content. Int J Obes (Lond). 2006;30(3):552-560.). The original sample size of the study was chosen to ensure a least detectable weight loss of 1.25-0.55 kg for an additive genetic model with the statistical power of 0.80 (PLoS Clin Trials. 2006;1(2):e12.). The dietary intervention was standardized to correspond to each participant’s daily energy requirement, as estimated from each participant’s measured basal metabolic rate, minus 2 512 kJ (600 kcal). The basal metabolic rate was measured at baseline by indirect calorimetry using ventilated hood systems. The mean caloric intake during intervention was thereby individual and corresponded to a mean of 6 183 kJ (1 477 kcal) for women and 8 066 kJ (1 927 kcal) for men, respectively. The participants went through examinations at baseline and after 10 weeks of follow-up including measures of body weight, height, waist- and hip circumference, bioimpedance measurements and blood sampling. |

**Supplemental Table 3. Outcome measurements**

| **Study name** | **Body weight (kg)** | **Waist circumference (cm)** | **Glycemic traits measurements** |
| --- | --- | --- | --- |
| **Abbreviation** | **Clinical measurement/ self-reported** | **Clinical measurement/ self-reported** |  |
| POUNDS Lost | Clinical measurement | Clinical measurement | Glucose and insulin were measured with the use of an immunoassay with chemiluminescent detection on an Immulite analyzer (Diagnostic Products Corporation). β cell function was estimated by homeostasis model assessment of β cell function (HOMA-B) with the use of the following equation: [20 × fasting insulin (μU/mL)]/{[fasting glucose (mg/dL)/18.01]−3.5}. Insulin resistance was estimated by HOMA-IR as follows: [fasting insulin (μU/mL)] × [fasting glucose (mg/dL)/18.01]/22.5 |
| DIETFITS | Clinical | Clinical | OGTT |
| DIOGENES | Clinical measurement | Clinical measurement | Fasting and OGTT serum glucose and insulin concentrations were analysed by a colorimetric assay (Ortho‐Clinical Diagnostics, Johnson & Johnson, Birkerød, Denmark) From the fasting glucose and the insulin concentrations, the HOMA-IR index, a measure of fasting insulin sensitivity, was calculated as: (fasting insulin (mIU/L) × fasting glucose (mmol/L))/22.5. Insulin sensitivity and β‐cell function indexes were calculated at fasting using the homeostasis model assessment for insulin resistance (HOMA‐IR) and HOMA for β‐cell function and quantitative insulin sensitivity check index |
| WAHA | Clinical measurement | Clinical measurement | Glucose (oxidase method adapted to a Siemens ADVIA2400), insulin (immunoassay adapted to a Siemens ADVIA Centaur). Beta-cell function by HOMA in the standard way |
| FinDPS or FDPS | Body weight annually measured | Annually measured | Glucose levels were measured locally by standard methods, and the measurements were standardized by the central laboratory. Serum insulin was determined with a radioimmunoassay (Pharmacia, Uppsala, Sweden) which shows 41% cross-reactivity with proinsulin.OGTT with glucose and insulin measurements, different insulin secretion and insulin resistance variables available. |
| PREDIMED-Reus | Clinical measurement | Clinical measurement | At baseline, blood samples were obtained from each participant after an overnight fast. Fasting glucose was measured using standard enzymatic automated methods |
| NUGENOB | Clinical measurement | Clinical measurement | Venous blood samples were drawn after an overnight fast of 12 h, following a 3-day period when subjects had been instructed to avoid excessive physical activity or alcohol consumption. Subjects rested in the supine position for 15 min before the procedure. Fasting plasma glucose was measured with standard enzymatic techniques on a COBAS FARA centrifugal spectrophotometer (Roche Diagnostica, Basel, Switzerland; glucose HK 125, ABX Diagnostics, Montpellier, France) Fasting plasma insulin concentration was measured with a double-antibody radioimmunoassay (Insulin, RIA 100, Kabi-Pharmacia, Uppsala, Sweden). All biochemical analyses were conducted independently of the allocated intervention groups in core facilities at the Department of Human Biology, Nutrition Research Centre NUTRIM, Maastricht University, and Medical Laboratories Dr Stein & colleagues, Mo¨nchengladbach, Germany. (Ref: Petersen M, Taylor MA, Saris WH, Verdich C, Toubro S, Macdonald I, Rossner S, Stich V, Guy-Grand B, Langin D, et al: Randomized, multi-center trial of two hypo-energetic diets in obese subjects: high- versus low-fat content. Int J Obes (Lond) 2006, 30:552-560.β cell function was estimated by homeostasis model assessment of β cell function (HOMA-B) with the use of the following equation: [20 × fasting insulin (μU/mL)]/{[fasting glucose (mg/dL)/18.01]−3.5}. Insulin resistance was estimated by HOMA-IR as follows: [fasting insulin (μU/mL)] × [fasting glucose (mg/dL)/18.01]/22.5 |

**Supplemental Table 4. Genotyping information in included studies.**

| **Study** | ***TCF7L2* (rs7903146), n(%)** | | | | |
| --- | --- | --- | --- | --- | --- |
|  | **CC** | **CT** | **TT** | **MAF** | **HWE p value** |
| DIETFITS | 213 (49%) | 139 (32%) | 82 (19%) | 0.35 | 0 |
| Diogenes | 381 (49.1%) | 329 (42.4 %) | 66 (8.5 %) | 0.3 | 0.67 |
| FinDPS | 212 (61.6%) | 114 (33.1%) | 18 (5.2%) | 0.22 | 0.6 |
| NUGENOB | 315 (51%) | 255 (41%) | 49 (8%) | 0.29 | 0.79 |
| POUNDS Lost | 291 (49.5) | 248 (42.2) | 49 (8.3) | 0.24 | 0.7 |
| PREDIMED-Reus | 302 (30.0%) | 391 (47.9%) | 124 (15.2%) | 0.39 | 0.88 |
| WAHA | 294(46.3%) | 274(43.1%) | 58(9.1%) | 0.31 | 0.61 |
| MAF=minor allele frequency for all randomized participants who provided genetic consent and whose DNA data passed quality control procedures; | | | | | |

**Supplemental Table 5. Covariates included in non-stratified linear regression models**

| **Study** | **Covariates** |
| --- | --- |
| POUNDS Lost | age (year), sex, ethnicity, body mass index at the baseline, each outcome-trait at the baseline, and diet/intervention groups. |
| DIETFITS | age (year), sex, current smoking habit (yes or no), physical activity habit (active or inactive), body mass index at the baseline, each outcome-trait at the baseline, and diet/intervention groups. |
| DIOGENES | age (year), sex, current smoking habit (yes or no), physical activity habit (active or inactive), body mass index at the baseline, each outcome-trait at the baseline, and diet/intervention groups. |
| WAHA | age (year), sex, ethnicity, current smoking habit (yes or no), physical activity habit (active or inactive), body mass index at the baseline, each outcome-trait at the baseline, and diet/intervention groups. |
| FinDPS or FDPS | age (year), sex, current smoking habit (yes or no), physical activity habit (active or inactive), body mass index at the baseline, each outcome-trait at the baseline, and diet/intervention groups. |
| PREDIMED-Reus | age (year), sex, current smoking habit (yes or no), physical activity habit (active or inactive), body mass index at the baseline, each outcome-trait at the baseline, and diet/intervention groups. |
| NUGENOB | age (year), sex, current smoking habit (yes or no), physical activity habit (active or inactive), body mass index at the baseline, each outcome-trait at the baseline, and diet/intervention groups. |

**Supplemental Table 6. Characteristics of studies included in qualitative synthesis (n=4114).**

| **Study** | **Number of participants** | **Type** | **Length, weeks** | **Ethnicity** | **Age, years** | **HWE p value** |
| --- | --- | --- | --- | --- | --- | --- |
| DIETFITS | <500 | Diet | ≥48 | White | <50 | <0.05 |
| Diogenes | ≥500 | Diet | <48 | White | ≥50 | >0.05 |
| FinDPS | <500 | Diet and exercise | ≥48 | White | ≥50 | >0.05 |
| NUGENOB | ≥500 | Diet | <48 | White | <50 | >0.05 |
| POUNDS Lost | ≥500 | Diet | ≥48 | Mixed* | ≥50 | >0.05 |
| PREDIMED-Reus | ≥500 | Diet | ≥48 | White | ≥50 | >0.05 |
| WAHA | ≥500 | Diet | ≥48 | White | ≥50 | >0.05 |
| PA: physical activity | | | | | | |
| *White (79%), black (16%), Hispanic (4%), other (1%). | | |  |  |  |  |

**Supplemental Table 7. Main genetic effect of TCF7L2 rs7903146 on changes in outcomes among all intervention groups (excluding control group)**

| **Changes in outcomes** | **Study name** | | | | | | | **Pooled** | | |
| --- | --- | --- | --- | --- | --- | --- | --- | --- | --- | --- |
|  | **DIETFITS** | **Diogenes** | **FinDPS** | **NUGENOB** | **POUNDS Lost** | **PREDIMED-Reus** | **WAHA** | **Beta±SE** | **Test for heterogeneity** | **Test for overall effect** |
| Body weight (kg) | -1.03±0.79 | -0.67±0.62 | 0.98±0.76 | 0.17±0.24 | -0.11±0.47 | -0.32±0.14 | -0.25±0.30 | -0.20±0.11 | X^2^=7.32 (d.f. = 6), p = 0.29, I^2^=18.0% | z=1.88 p=0.061 |
| Waist circumference (cm) | -1.78±1.03 | 0.05±0.67 | 0.03±0.77 | 0.34±0.30 | -0.02±0.51 | -0.74±0.21 | -0.44±0.34 | -0.36±0.14 | X^2^=11.63 (d.f.=6), p=0.07, I^2^=48.4% | z=2.55 p=0.011 |
| Fasting glucose (mmol/L) | 0.50±0.96 | 0.09±0.06 | -0.04±0.08 | -0.01±0.03 | -0.62±0.62 | 0.01±0.08 | -1.55±1.38 | 0.01±0.02 | X^2^=5.32 (d.f.=6), p=0.50, I^2^= 0.0% | z=0.33 p=0.138 |
| Fasting insulin (mIU/L)* | -0.02±0.05 | -0.04±0.02 | -0.06±0.03 | 0.04±0.04 | 0.00±0.03 | / | 0.00±0.05 | -0.02±0.01 | X^2^=4.97 (d.f.=5), p=0.42 ,I^2^= 0.0% | z=1.64 p=0.101 |
| HOMA-IR* | -0.02±0.05 | -0.03±0.03 | -0.06±0.03 | 0.02±0.04 | -0.01±0.03 | / | 0.02±0.06 | -0.02±0.01 | X^2^=3.25 (d.f.=5), p=0.66, I^2^= 0.0% | z=1.42 p=0.156 |
| HOMA-B* | -0.03±0.05 | -0.05±0.02 | -0.05±0.02 | 0.03±0.04 | 0.03±0.02 | / | -0.03±0.05 | -0.02±0.01 | X^2^=10.45 (d.f.=5), p=0.03, I^2^=52.2% | z=1.50 p=0.132 |

*Data on insulin, HOMA-IR and HOMA-B are log-transformed before analysis

Values represent β coefficient and standard error for change in body weight, waist circumference and glycemic traits by *TCF7L2* minor allele (allele-dose model was employed and coded in terms of copies of minor allele (0, 1, 2)).

**Supplemental Table 8. Stratified analyses to identify potential moderators of relation between TFC7L2 rs7903146 genotype and intervention effects on change in outcomes**

| **Variables** | **Fasting glucose (mmol/L)** | | **Fasting insulin (mIU/L)*** | | **HOMA-IR*** | | **HOMA-B*** | | **Body weight (kg)** | | **Waist circumference (cm)** | |
| --- | --- | --- | --- | --- | --- | --- | --- | --- | --- | --- | --- | --- |
|  | **Mean difference** | **P value** | **Mean difference** | **P value** | **Mean difference** | **P value** | **Mean difference** | **P value** | **Mean difference†** | **P value** | **Mean difference** | **P value** |
| **No. of participants** |  |  |  |  |  |  |  |  |  |  |  |  |
| <500 (n=2) | 0.48(-0.56,1.51) | 0.366 | -0.03(-0.05,0.00) | 0.041 | -0.03(-0.07,0.01) | 0.124 | -0.03(-0.03,-0.02) | <0.001 | 0.43(-1.06,1.92) | 0.572 | 0.23(-1.26,1.71) | 0.764 |
| ≥500 (n=5) | -0.42(-0.63,-0.21) | <0.001 | 0.01(-0.06,0.08) | 0.765 | 0.02(-0.06,0.10) | 0.643 | 0.00(-0.06,0.06) | 0.997 | 0.15(-0.41,0.70) | 0.6 | 0.10(-0.48,0.67) | 0.742 |
| **Intervention:** |  |  |  |  |  |  |  |  |  |  |  |  |
| Diet (n=6) | -0.20(-0.40,0.00) | 0.046 | 0.00(-0.05,0.06) | 0.863 | 0.01(-0.05,0.07) | 0.69 | -0.01(-0.05,0.04) | 0.745 | 0.07(-0.43,0.56) | 0.785 | -0.01(-0.52,0.50) | 0.976 |
| Diet/exercise (n=1) | -0.05(-0.06,-0.03) | <0.001 | -0.04(-0.04,-0.03) | <0.001 | -0.05(-0.06,-0.04) | <0.001 | -0.03(-0.03,-0.02) | <0.001 | 1.19(1.02,1.37) | <0.001 | 0.99(0.81,1.16) | <0.001 |
| **Study length:** |  |  |  |  |  |  |  |  |  |  |  |  |
| <48 weeks (n=2) | -0.29(-0.60,0.00) | 0.046 | -0.03(-0.04,-0.01) | 0.006 | -0.02(-0.04,0.01) | 0.116 | -0.03(-0.05,-0.01) | 0.018 | -0.07(-0.35,0.50) | 0.731 | -0.01(-0.58,0.59) | 0.975 |
| ≥48 weeks (n=5) | 0.05(-0.02,0.11) | 0.19 | 0.01(-0.12,0.14) | 0.888 | 0.01(-0.14,0.17) | 0.865 | 0.00(-0.08,0.09) | 0.945 | 0.64(0.32,0.97) | <0.001 | 0.49(0.42,0.56) | <0.001 |
| **Age (years):** |  |  |  |  |  |  |  |  |  |  |  |  |
| <50 (n=2) | 0.54(-0.37,1.45) | 0.247 | 0.03(-0.06,0.12) | 0.483 | 0.04(-0.06,0.14) | 0.422 | 0.01(-0.06,0.08) | 0.801 | 0.23(-0.86,1.31) | 0.683 | -0.01(-1.02,0.99) | 0.978 |
| ≥50 (n=5) | -0.45(-0.67,-0.23) | <0.001 | -0.04(-0.06,-0.03) | <0.001 | -0.04(-0.06,-0.02) | 0.001 | -0.03(-0.06,-0.01) | 0.011 | 0.23(-0.22,0.67) | 0.323 | 0.19(-0.41,0.80) | 0.532 |
| **HWE p value** |  |  |  |  |  |  |  |  |  |  |  |  |
| ≤0.05 (n=1) | 1.01(0.82,1.21) | <0.001 | -0.01(-0.02,0.00) | 0.005 | -0.01(-0.02,0.00) | 0.037 | -0.03(-0.04,-0.02) | <0.001 | -0.33(-0.48,-0.18) | <0.001 | -0.53(-0.71,-0.35) | <0.001 |
| >0.05 (n=6) | -0.33(-0.49,-0.17) | <0.001 | 0.00(-0.05,0.05) | 0.954 | 0.00(-0.05,0.06) | 0.894 | -0.01(-0.05,0.03) | 0.703 | 0.32(-0.21,0.85) | 0.233 | 0.24(-0.30,0.79) | 0.377 |

*Data on insulin, HOMA-IR and HOMA-B are log-transformed before analysis

Values represent coefficient and 95% confidence interval for changes in outcomes after weight loss intervention for each copy of the *TCF7L2* (rs7903146) minor allele T (allele-dose model was employed and coded in terms of copies of minor allele (0, 1, 2)) in treatment versus control arm in random effects meta-analysis of 4114 adult.
